# Community caregivers and innovative financing: a qualitative analysis of Thailand's Ubolratana Hospital model using the consolidated framework for implementation research

**DOI:** 10.1093/heapol/czag091

**Published:** 2026-07-16

**Authors:** Sila Tonboot, Pantitra Singkheaw, Attakorn Raksasataya, Noppawan Poonpipat, Sirilakkhana Phrawong, Watcharapong Rintra, Charoonrat Srisespimp, Wiroj Wannapira, Supasit Pannarunothai

**Affiliations:** Centre for Health Equity Monitoring Foundation, 271 Moo 6, Thapho, Mueang, Phitsanulok 65000, Thailand; Department of Family Medicine, Faculty of Medicine, Naresuan University, 99 Moo 9, Thapho, Mueang, Phitsanulok 65000, Thailand; Karunruk Palliative Care Center, Faculty of Medicine, Khon Kaen University, 123 Mittraphap Road, Mueang, Khon Kaen 40002, Thailand; Centre for Health Equity Monitoring Foundation, 271 Moo 6, Thapho, Mueang, Phitsanulok 65000, Thailand; Department of Social Medicine, Khon Kaen Hospital, 54 Sri Chan Road, Mueang, Khon Kaen 40000,Thailand; Department of Palliative Care, Khon Kaen Hospital, 54 Sri Chan Road, Mueang, Khon Kaen 40000, Thailand; Department of Palliative Care, Khon Kaen Hospital, 54 Sri Chan Road, Mueang, Khon Kaen 40000, Thailand; Buddhachinaraj Phitsanulok Hospital, 90 Srithammatripidok Road, Mueang, Phitsanulok 65000, Thailand; Centre for Health Equity Monitoring Foundation, 271 Moo 6, Thapho, Mueang, Phitsanulok 65000, Thailand

**Keywords:** community, primary healthcare, resource allocation, health policy

## Abstract

Thailand’s healthcare system faces challenges in delivering effective community-based care for dependent patients in rural areas; while access has expanded over more than four decades, services remain fragmented and effective coverage from community to higher-level care is still lacking. The Ubolratana Hospital model in Khon Kaen Province represents an innovation, combining a full-time 24-hr community caregiver cadre integrated within a hospital-led local healthcare team with five adaptive diversified funding streams. This qualitative implementation research, guided by the Consolidated Framework for Implementation Research (CFIR), examined the determinants of adoption and sustainment of the model over its 6-year operation. The CFIR-informed interview guide was used as the key data collection and analytical framework across its five domains [innovation, outer setting, inner setting, individuals (roles and characteristics), and implementation process]. We conducted semi-structured interviews with key informants spanning hospital administrators, physicians, nurses, community caregivers, sub-district primary care unit personnel, local government officials, community leaders, and family members between March and May 2025. Data were analysed using framework analysis combining deductive CFIR coding with inductive sub-coding. Adoption was facilitated by a hospital-initiated caregiver role, sustained inner-setting leadership embodied in a long-tenured ‘social engineer’ hospital director and his successor team, a three-stage community-led caregiver selection process, and Buddhist-integrated spiritual care; sustainment relied on a coordinated multi-level care process and the continuity of care centre’s reflective evaluation. Outer-setting barriers included fragmented public health financing, the 2022 decentralization of sub-district primary care units to provincial administrative organizations, and donor-funding uncertainty. Since 2020 the model has been progressively scaled under Ramathibodi Faculty of Medicine sponsorship to 22 districts across 15 provinces (53 villages, cumulative investment >10 million baht). The Ubolratana experience therefore offers a practical, rather than directly replicable, framework whose diffusion depends on cultivating local leadership, building trust, and adapting innovation components to local conditions.

Key messages

- Rural community-based care in Thailand faces effectiveness gaps despite expanded access; this study uses the Consolidated Framework for Implementation Research to analyse how one community hospital initiated and sustained a community-caregiver model for 6 years.
- Adoption and sustainment depended on inner-setting leadership, hospital-team integration, and implementation-process design (particularly coordinated multi-level care and a continuity of care centre) more than on financing mechanisms alone.
- Outer-setting determinants (fragmented financing and post-decentralization governance) remain the principal barriers to wider replication in Thailand’s decentralized health system.
- For policymakers, the model offers a practical framework, demonstrating that diffusion is a feasible framework for scale-up, enforcing leadership development and community trust building.

## Introduction

Thailand’s healthcare system faces persistent challenges in delivering effective community-based care for dependent patients and people with chronic or end-of-life needs in rural areas despite successful universal health coverage (UHC) implementation (Rojjananukulpong et al. 2021). While access to primary care has expanded over more than four decades, services remain fragmented and effective coverage, from community to higher-level care, is still lacking; the present paper documents one local effort to close that gap through a task-specific caregiver cadre rather than a generic expansion of primary care. The 2022 decentralization of sub-district health-promoting hospitals (later called primary care units or PCUs) to provincial administrative organizations (PAOs) has facilitated opportunities for enhancing healthcare delivery through improved service quality and nursing competencies, while placing greater pressure on community hospitals to address resource constraints and maintain efficiency (Sriyasak et al. 2025). The Ubolratana Hospital model in Khon Kaen Province represents an innovative, locally initiated response to these challenges through hospital integrated community caregivers and diversified funding mechanisms that redefine how rural healthcare teams reach dependent patients at home.

Thailand’s community hospital system has been a key advocate of UHC (Ramon Magsaysay Award Foundation 2024). Many community hospital directors, or rural doctors serve as ‘social engineers’, extending their leadership beyond hospital managment to mobilise district health system actors and address the broader social determinants of health (Mahidol University 2023). At Ubolratana Hospital, Apisit Thamrongwarangkun (a 1983 Ramathibodi Hospital MD graduate, retired after serving as long-term director) has led innovations in caregiver training, building up an informal long-term care (LTC) workforce in the community of Ubolratana district. The innovations mobilize natural resources through community banking mechanisms (organic foods, bananas, bamboos, planted trees), reorient deficit and disability into power potentials (the ‘change burden into power’ campaign), and partner with business sectors (Dream World leisure park and the Siam Cement Group Foundation). This established leadership and proven sustainability positioned Ubolratana as the ideal study site for examining resource management in rural contexts.

Unlike traditional models that rely primarily on government funding and conventional healthcare workers and village health volunteers or part-time LTC caregivers (Kowitt et al. 2015), the Ubolratana model initiated a full-time community caregiver role for task-specific community care. While the National Health Security Office’s caregivers, working part-time, focus on basic care activities for bed-bound patients (National Health Security Office 2024), Ubolratana’s community caregivers provide 24-hr home-based support for chronic disease follow-up, prevention, basic assessment, palliative care, and acute-care coordination under hospital supervision. Critically, they are integrated as members of the local hospital-led healthcare team rather than as an independent or parallel community health worker (CHW) cadre, which preserves continuity rather than adding a new service layer.

The financial sustainability challenge that typically limits community-based healthcare programmes is addressed through adaptive innovative financing strategies, including community contributions, local government and National Health Security Office (NHSO) streams, social-enterprise revenue, and partnerships with private sectors, as seen in broader discussions of community health workers in low- and middle-income countries (Mishra et al. 2019). In this paper, innovative financing is treated as an enabling mechanism for sustaining and scaling the caregiver innovation, rather than as the sole innovation. The reliance on multiple funding streams also highlights systemic inefficiencies in Thailand’s public health financing, characterized by fragmented budget allocation. Additionally, the decentralization of PCUs to PAOs has complicated governance and oversight, creating challenges in coordinating community-based care.

Using the Consolidated Framework for Implementation Research (CFIR) as both an interview-sensitizing framework and an analytical determinant framework (Damschroder et al. 2022, Reardon et al. 2025), we examine the barriers and facilitators of adoption and sustainment of this 6-year-old model. Our research question was: What CFIR-defined determinants explain the adoption and sustainment of the Ubolratana Hospital model, and what implications does this hold for replication elsewhere in Thailand’s decentralized health system?

## Methods

### Study design and theoretical framework

This qualitative implementation research was conducted as part of a larger project examining appropriate community end-of-life care after the Ministry of Public Health began transferring sub-district PCUs to PAOs. The CFIR was used in two related ways: first, as a sensitizing framework for the interview guide development so that questions covered innovation, outer setting, inner setting, individuals, and implementation process issues; and second, as the key analytical framework for identifying barriers and facilitators (CFIR determinants) of two implementation outcomes (adoption and sustainment) of the Ubolratana model over its 6-year operation (Damschroder et al. 2022, Reardon et al. 2025).

### Setting

The study was conducted in Ubolratana District, Khon Kaen Province, northeastern Thailand, covering ∼40 004 residents across 12 000 households served by Ubolratana Hospital (a 60-bed community hospital) and eight sub-district PCUs.

### Participants and sampling

Participants were selected through purposive sampling to include key stakeholders: hospital administrators (*n* = 2), physicians (*n* = 5), nurses (*n* = 3), community caregivers (*n* = 3), sub-district PCU personnel (*n* = 2), local government officials (*n* = 2), and community leaders with patient family members (*n* = 3).

### Data collection

Semi-structured in-depth interviews were conducted between March and May 2025 using a CFIR-informed interview guide. The guide was not administered as rigid domain labels, but questions were designed to elicit data across all five CFIR domains: how the caregiver model differed from existing services and was adapted (innovation); how financing, policies, decentralization, and partnerships shaped implementation (outer setting); how hospital culture, leadership, resources, and any other contextual factors influenced the model (inner setting); how leaders, caregivers, patients, families, PCU staff, and community actors experienced their roles (individuals); and how selection, training, coordination, supervision, and reflective evaluation was performed (implementation process). Interviews, lasting 60–90 min each, were conducted in Thai by trained researchers, audio-recorded with participant consent, and transcribed verbatim with field notes capturing contextual information.

### Data analysis

Analysis followed a framework approach that combined deductive CFIR coding with inductive sub-coding (Reardon et al. 2025), informed by Braun and Clarke's six-phase thematic analysis (Braun et al. 2023): data familiarization, initial coding, theme searching, reviewing and refining, defining and naming, and report writing with illustrative quotes. Two researchers (S.T. and N.P.) independently coded the data, with discrepancies resolved through discussion and consensus. Codes were mapped to CFIR constructs through framework analysis, while inductive sub-codes were retained when interviewees raised issues not explicitly anticipated in the guide. For example, Buddhist spiritual integration was coded primarily under inner setting (culture and mission alignment) and cross-referenced to outer-setting partnerships where the temple functioned as an external partner. Thai quotes were translated into English preserving meaning and context. The coded data underlying this article will be shared on reasonable request to the corresponding author.

### Ethics

The study received institutional review board approval from the Institute for Development of Human Research Protection (IHRP), Health Systems Research Institute (HSRI), with Certificate of Approval (COA) No. IHRP2024176 and IHRP No. 119-2567. Written informed consent was obtained from all participants, ensuring confidentiality through the use of pseudonyms for non-public figures. Roles such as hospital administrators and key donors were identified by position with their consent. Additional policy insights were derived through researcher analysis, integrating findings with broader health system contexts.

## Results

We present findings organized by the five CFIR domains, followed by a section on cross-cutting barriers. Within domains, findings are organized to distinguish enablers/supporters from barriers/challenges where appropriate. Table 1 summarises the determinants identified across domains; the narrative below details each determinant with illustrative quotations from informants. Where determinants span more than one domain, we discuss them in the primary domain and cross-reference.

**Table 1:** CFIR domains, determinants identified at Ubolratana district, and their facilitator or barrier valence for adoption and sustainment.

CFIR domain	Key determinant(s) at Ubolratana	Valence (adoption/sustainment)
Innovation	Innovation design: caregiver curriculum, embedded team role, route-mapped caseload, multi-stream financing architecture. Adaptability: caregiver role reshaped over time, funding streams added incrementally. Complexity: moderately high (training, supervision, accounting infrastructure). Innovation evidence base: 6-year continuous operation; service outcomes (HbA1c control, wound healing, end-of-life uptake) interpreted as innovation outcomes rather than design quality	Facilitator (both)
Outer setting	Financing: fragmented public health financing; double-tax-deductibility donation channel under State Audit Office oversight. Policies and Laws: post-2022 decentralization to PAOs; Section 35 of the Persons with Disabilities Empowerment Act. Partnerships and Connections: principal private donor (a Bangkok-based amusement-park owner), Siam Cement Group Foundation, seven sub-district administrative organizations, and a local Buddhist temple hosting the hospice monastery. Local attitudes: Buddhist-integrated spiritual care	Mixed (financing fragility, barrier; partnerships, facilitator)
Inner setting	Culture and mission alignment: ‘social engineer’ framing of clinical staff. Structural characteristics: 60-bed hospital with 10 attending physicians; COC centre; food market for chemical-free produce; supply-chain optimization. Communications and relational connections: shared digital channels and case discussions across caregiver–physician–COC teams. Tension for change: workforce shortages and post-decentralization flux	Facilitator (both)
Individuals (roles and characteristics)	High-level leader and implementation lead/champion (both roles converging on the former long-term hospital director). Co-implementation lead: director of a neighbouring community hospital. External change ggent: senior Thai public-health scholar (intellectual progenitor). Innovation deliverers: caregivers (hybrid farmer–clinician identity). Innovation recipients: patients and families. Other implementation support: village headmen, Buddhist monks, family members structurally embedded in selection	Strong facilitator (adoption); transition risk (sustainment)
Implementation process	Teaming and planning (three-stage selection); engaging innovation deliverers and assessing context and needs and tailoring strategies (the same selection process); teaming and doing (the ‘three doctors’ coordinated-care model, supported by inner-setting communications and relational connections); doing (route-mapped daily caseload, 10–15 patients/day); reflecting and evaluating (COC-based outcome tracking with HbA1c 8%–9% cohort quality-improvement loop); adapting (incremental adaptation over a decade)	Facilitator (both)
Cross-cutting barriers	Donor funding sunset; role confusion (caregiver/VHV/LTC); post-decentralization governance gap; scalability cost; quality-assurance scope creep; rising community expectations	Barriers

### Domain 1. Innovation: a 24-hr hospital-integrated caregiver cadre and diversified innovative funding streams

The Ubolratana innovation is best understood as a locally initiated caregiver model rather than an adopted package. Its design logic began with the unmet need of dependent patients, people with chronic conditions requiring palliative or end-of-life care. The first design element is a redefined community caregiver cadre: full-time, available 24 hr/day for 6 days/week, selected through hospital-team screening and community endorsement, employed and supervised through the hospital-led care team, and trained through a 6-month enhanced-competency-based curriculum delivered jointly with a partner academic institution. The programme combines Monday-to-Friday theoretical instruction online with Saturday supervised field practice in the trainee’s home village, totalling ∼720 hr of training (compared with the standard 70-hr LTC caregiver curriculum), covering medication administration, diabetes management, basic diagnostic procedures, palliative care, emergency triage, documentation, and team communication. Trainees receive a 300-baht daily stipend from the first day of training to enable participation among candidates who could not otherwise afford to leave subsistence work for 6 months. Critically, these caregivers are positioned as integral members of a hospital-led local healthcare team rather than as independent minimally paid volunteers or a parallel CHW cadre. A community caregiver described the transformation:

‘Before training, I was just a farmer who cared about my neighbours. During the training at the hospital, I learned to provide comprehensive care, undertook diagnostic procedures, managed diabetes medications, and even assisted in emergency situations. We rotated through different departments, emergency room, medical and paediatric wards, and palliative care. By the end, I felt confident handling most health problems in my village.’

The motivation for this redefined role emerged from systematic gap analysis. A hospital administrator described the reasoning:

‘When we analyzed the traditional caregiver system, we found significant gaps. LTC caregivers would visit briefly, provide basic care, and leave. There was no continuity, no prevention, no health education. We saw the need for someone who could live in the community, understand the people, and provide comprehensive healthcare services 24 hr a day.’

A sub-district PCU staff member confirmed the operational difference 24-hr availability makes:

‘When we need someone urgently, we can call the community caregiver directly. They have the knowledge and skills to handle situations that would typically require a nurse. This saves precious time, especially in emergencies.’

Operationally, each caregiver visits 10–15 patients daily on a weekly cycle, with morning routes (8–10 a.m.) typically covering 8–10 patients (occasionally up to 15) clustered along shared streets, a deliberate route-mapping innovation introduced to reduce travel time across villages that can span 4–5 km between dwellings.

The second component is a five-stream funding model, an adaptive financing architecture that enables the caregiver role to be sustained. The model combines (i) hospital self-financing, (ii) NHSO primary care budget, (iii) community contributions through agricultural products, (iv) social enterprise revenue (including Section 35 of the Persons with Disabilities Empowerment Act enforced for big facilities employing persons with disabilities), and (v) local government partnerships, and private philanthropy from Dream World leisure park, which initially funded five caregivers each in five pilot districts (Dan Sai and Lom Kao in Phetchabun province, Khun Han in Si Sa Ket, and Nam Phong and Ubolratana in Khon Kaen) at ∼80 000 baht per caregiver per year, for a 5-year period subsequently extended by a further year. The size of contribution of each stream was not fixed; interviewees described how everything keeps changing from year to year. Donations were channelled through the hospital’s operating-funds account under an arrangement (co-designed by the partner community-hospital director and approved during the tenure of a former Minister of Public Health) that confers double tax deductibility on donors while bringing the funds under State Audit Office oversight. An academic partnership with a training institution provides the theoretical and practical training components. A hospital administrator framed the agricultural component:

‘Each bamboo clump produces 10–50 shoots annually. A fresh shoot sells for 10–20 baht, but the value of a processed shoot increases to 50–100 baht. If a community of 100 households each donate one shoot per year, we can generate sustainable funds from agricultural promotion.’

The model showed strong innovation adaptability: the caregiver role was reshaped over time, and funding streams were added incrementally as opportunities arose. Innovation complexity is moderately high because the model requires sophisticated training, handling of complex care, case documentation, consultation, supervision, accounting, and trust-building infrastructure. Innovation design is reflected in the 24-hr caregiver role, the enhanced-competency-based curriculum, the embedded team role, the route-mapped daily caseload, the community-endorsed selection process, and the adaptive multi-stream financing architecture. A plausible evidence base for the innovation is supported by 6 years of continuous operation and subsequent scale-up to other districts. Service outcomes consistently exceeded national targets, including achievement of the haemoglobin A1c (HbA1c) < 7% control target of at least 40% (a target Ubolratana district had not previously achieved in any year), improved wound healing, and 95% home-based end-of-life-care uptake (Tonboot et al., 2026).

Since 2020 the model has progressively scaled through institutional sponsorship by the Faculty of Medicine, Ramathibodi Hospital, Mahidol University. A five-pillar programme [covering (i) hospital service development, (ii) community health systems and caregiver workforce, (iii) hospital financial systems, (iv) workforce in scarce specialties, and (v) education], extended the initial 3-year funding (2020–2022) with a further 2 years (2023–2024). The model now operates in 22 districts across 15 provinces (53 villages), with cumulative investment >10 million baht (Pannarunothai et al. 2025). Original pilot districts received five caregivers each; later sites received two. This expansion constitutes a substantive test of the ‘practical framework’.

### Domain 2. Outer setting: policy constraints, financing barriers/enablers, and external partnerships

Outer-setting determinants shaped both adoption and sustainment as enablers/supporters and barriers/challenges. First, policies and laws created a major challenge: the 2022 transfer of sub-district PCUs to PAOs reconfigured governance and accountability, creating district-to-district variation in whether hospitals could continue supervising and funding community caregivers. A hospital director highlighted the resulting oversight gap:

‘Inconsistent policies due to changes in local leadership make it difficult to supervise caregivers effectively, as hospitals are no longer the direct payers.’

Section 35 of the Persons with Disabilities Empowerment Act enabled Ubolratana hospital to employ 24 workers with disabilities, generating corporate-partnership revenue and social value simultaneously. A hospital administrator described how this mechanism operates in practice:

‘We employ 24 people with various disabilities for various roles, housekeeping, gardening, food preparation, medical record keeping, and community outreach. Some companies pay for salaries of these positions at our hospital rather than hiring directly at their companies.’

Second, financing functioned as both an enabler and a barrier. Multiple funding sources created resilience against shocks in any one stream, but fragmented budgets, donor uncertainty, and administrative complexity also constrained scale-up. This outer-setting financing constraint is the reason the adaptive financing architecture is described under Domain 1 as an enabling mechanism for the innovation. A PCU director explained how the model exploits NHSO funds as a deliberate implementation-process revenue strategy:

‘We realized that NHSO pays for certain primary care services that community caregivers can provide more cost efficiently than hospital-based staff. By training our caregivers to document and claim these services properly, we can maximize revenue while providing better patient access.’

Third, partnerships and connections with Dream World leisure park, Siam Cement Group Foundation, seven sub-district administrative organisations, a local Buddhist temple (which hosts the programme’s hospice monastery), and academic partners underpinned both initial adoption and continued sustainment. These partnerships supplied training capacity, equipment, and local legitimacy, while their instability also exposed the programme to sustainability risk. The local Buddhist temple is also discussed in Domain 3 inner setting because interviewees described it primarily as part of the district’s cultural and spiritual care. A hospital administrator acknowledged the vulnerability of this outer-setting determinant:

“Our main donor’s commitment is ending, and we’re uncertain about renewal. While we’ve developed multiple funding streams, the loss of any major source could threaten programme continuity.”

### Domain 3. Inner setting: social engineer leadership, Buddhist cultural integration, and available resources

The inner setting of Ubolratana Hospital is characterized by culture, i.e. mission alignment leadership that frames clinical staff as ‘social engineers’ for the district health system. Apisit Thamrongwarangkun, the hospital’s former long-term director, was central to developing this culture, but interviewees also described its continuity into the next leadership generation, with the current director and hospital team carrying forward the same community-development orientation. This succession suggests that leadership is not simply transferable as a technical component, but it can be deliberately built through mentorship, institutional culture, and long-term local ownership. Structural characteristics include a 60-bed community hospital with 10 attending physicians, a continuity of care (COC) centre that systematically coordinates discharge planning, and supply-chain optimization that reduces medication waste. The hospital also operates a ‘Food Market’ that purchases pesticide-free produce from local farmers (generating both community income and chemical-free ingredients for inpatient meals) and uses room-naming-fundraising for corporate and community donations of ≥200 000 baht to convert episodic philanthropy into long-running available resources. A hospital administrator articulated the underlying logic:

‘We tracked the data. Patients with good community care had fewer emergency admissions, shorter hospital stays, and better treatment compliance. The calculated cost savings from preventable complications far exceeded our investment in community caregivers.’

Relational connections inside the hospital are strong: caregivers, physicians, and COC staff use shared digital channels and case discussions, enabling rapid coordination. Access to knowledge and information is supported by ongoing training and external academic partnership with a local college. Tension for change is acute: ongoing workforce shortages and post-decentralization flux create internal motivation to develop alternative cadres. Buddhist cultural integration extends over the hospice monastery at a local temple and functions as part of the district’s inner setting because it shapes how families understand illness, dying, caregiving, and trust in the hospital-led model. A Buddhist monk involved with the programme described the complementarity:

‘When families face serious illness or approaching death, they need both medical care and spiritual support. I work closely with the community caregivers to provide comfort and guidance based on Buddhist teachings. This isn’t separate from medical care, it’s complementary.’

### Domain 4. Individuals (roles and characteristics): caregiver self-efficacy, recipients, and community actors

At the individual level, implementation depended on the roles and characteristics of innovation deliverers, recipients, and community actors who made the model workable in daily practice. Former and current hospital leaders are discussed under Domain 3 because interviewees framed leadership primarily as part of the hospital’s inner setting culture. Domain 4 therefore focuses on caregivers’ self-efficacy after training, recipients’ trust and expectations, and the roles of village headmen, Buddhist monks, family members, PCU nurse supervisors, local government officials, donors, and academic partners as actors influencing implementation. Several participants emphasised that the model cannot be reduced to technical components; it depends on whether these actors recognise the caregiver as a competent, accountable member of the hospital-led team.

Innovation deliverers (the community caregivers themselves) exhibit a hybrid identity (farmer + clinical worker) that reinforces community trust, and the 6-month competency-based training was repeatedly described as producing self-efficacy, confidence, and skill. A caregiver described the 24-hr availability that distinguishes their role:

‘Last week, I received calls at 2 a.m. and 6 a.m. for different emergencies. In one case, a diabetic patient was having severe hypoglycaemia. I guided the family through emergency care, coordinating with the hospital for patient transport. This immediate response can save lives.’

Innovation recipients (patients and families) were initially sceptical of caregivers without formal medical credentials, as exemplified by the mother of a young woman with severe brain injury:

‘Her mother said to me, “Why should I trust a village person with my daughter's life? You don’t have medical training”.’

Six months of intensive home rehabilitation (physical therapy, respiratory care, nutritional support, family counselling) raised the patient’s Barthel (activities of daily living) index from 0 to 14–15. The community caregiver who led the case summarised the outcome:

‘Today, she can walk independently, ride a bicycle, feed herself, and relearn computer skills for potential employment. Her ADL scores improved from 0 to 14–15. The hospital team was amazed and refers similar cases to our programme.’

Other individuals (village headmen, Buddhist monks, family members, PCU multidisciplinary supervisors, local government officials, donors, and academic partners) operate as community stakeholders whose endorsement, supervision, and resource mobilization are structurally embedded in the selection, training, and sustainment process.

### Domain 5. Implementation process: community participation, coordinated multi-level care, and second-loop learning

Four implementation process determinants distinguish the Ubolratana model. First, the three-stage caregiver selection process operationalizes community participation and several CFIR sub-processes simultaneously: teaming and planning (assembling the team that will deliver the innovation), engaging innovation deliverers (recruiting the right candidates), assessing context and assessing needs (testing whether the candidate fits the village’s social fabric and trust environment), and tailoring strategies (matching person to community). Because this selection process also defines the caregiver role, it straddles innovation and implementation process domains. A hospital administrator described the underlying philosophy:

‘We learned earlier that external selection didn’t work. Community members need to trust their caregiver with absolute confidence, they confide their health, their family secrets, their fears about death and disability to their caregiver. This trust can only come when the community members choose their own representative.’

A successful candidate described the three stages verbatim:

‘First, I had to prove I was a genuine farmer by successfully growing bananas for a full season. Then the community held a public meeting where everyone could ask questions and raise concerns. Finally, community leaders, including the village headman, VHV (village health volunteer) leaders, sub-district PCU staff, and the local Buddhist monk, formally endorsed my candidacy at the Ubolratana hospital.’

A community caregiver described the cultural weight that this process generates:

‘Because the whole village participated in choosing me, they feel responsible for my success. When I need help organizing health activities or convincing families to change unhealthy behaviours, community members support me because they see me as their representative, not an outsider imposed by the hospital.’

This three-stage process is, in the team’s account, a deliberate response to the progressive erosion of earlier community-worker models in Thailand. Village health volunteer (VHV) compensation rose over a decade from 600 to 1000 and now to >2000 baht per month, but hospital informants estimated that only 30%–40% of VHVs function actively, with the proportion lower where local management is weaker. An earlier 6000-baht-per-case part-time caregiver scheme generated fairness disputes, paying inactive caregivers the same as active ones. The Ubolratana model’s full-time, community-validated cadre was designed as a corrective to both decay paths. A further procedural innovation is that each trainee undertakes the practical component at the hospital nearest to her or his own residence rather than at Ubolratana, establishing the local clinical relationships needed for safe complex care (e.g. when a tube becomes dislodged, the trainee already knows who to call) and reducing legal exposure for procedures sitting at the edge of nurse-aide scope.

The Ministry of Public Health’s ‘Three Doctors’ model (Sam Moh in Thai) is clearly translated into a coordinated multi-level care process linking (i) community-level caregivers, to (ii) sub-district PCU staff, and (iii) family physician at Ubolratana hospital for proper clinical care pathways. In CFIR terms, this is an implementation process: teaming and doing, supported by inner-setting communications and relational connections, with smartphone-based telemedicine, secure messaging, and Bluetooth-connected monitoring devices contributed by a corporate partner (Noree et al. 2022). A hospital administrator explained the inverted logic of the model:

‘Traditional healthcare assumes that patients should go to see doctors. But in rural areas, this advice creates barriers, transportation costs, time away from work, family obligations. We reversed this advice by bringing healthcare expertise to the community while maintaining professional oversight.’

A community caregiver illustrated systematically achieving a higher level of practice:

‘Last month, I had a diabetic patient with chronic diabetic foot. I took photos and sent them to doctor 2 via our communication system. She consulted with doctor 3, and together we developed a treatment plan. I provided daily wound care, doctor 2 monitored progress through photos, and doctor 3 adjusted medications via telehealth. The wound healed without hospitalization.’

Reflecting and evaluating is operationalized through the COC centre, which tracks outcomes systematically and converts routine service data into ongoing programme adjustment (National Health Security Office 2024). A COC staff member described the discharge-coordination process:

‘When a patient is about to be discharged from our hospital, we coordinate with their particular community caregiver, sub-district PCU, and family to ensure they have everything needed for successful recovery. We also maintain ongoing communications to minimize preventable readmission.’

Reflecting, evaluating, and adapting operate as second-loop learning rather than simple monitoring. The team audits patients with HbA1c in the 8%–9% range at the start of the fiscal year (rather than waiting for the year-end retrospective), targets them for intensive follow-up, reviews progress at 3 months, and reallocates caregiver time toward newer cases if patients have not converged on target, a ‘most-movable-first’ triage that traded universal control for cohort-level achievement of the at least 40% benchmark. Adaptation has occurred incrementally over more than a decade, with the caregiver role and funding streams evolving in response to local conditions and feedback.

### Cross-cutting barriers

Six barriers cut across or sit within CFIR domains and complicate both sustainment and replication: (i) financial uncertainty from impending donor sunset (outer setting: financing); (ii) role confusion between caregivers, VHVs, and LTC caregivers that occasionally generates conflict with other healthcare providers (individuals, with implications for implementation process); (iii) the post-decentralization governance gap, as hospitals no longer directly control fund flows to caregivers (outer setting: policies and laws, with inner-setting governance implications); (iv) scalability constraints, intensive training, community selection, and funding development require significant initial investment and time to develop (implementation process); (v) quality assurance and scope-creep risks as experienced caregivers occasionally exceed appropriate boundaries (implementation process: reflecting and evaluating); and (vi) evolving community expectations that strain caregiver availability (innovation recipients: patients and families). A community caregiver captured the success paradox:

‘Families now expect me to solve all their health problems immediately. They call for minor issues that they used to handle themselves, and they want me available constantly. Managing these expectations while maintaining boundaries is becoming more difficult.’

Role confusion was specifically reported by a sub-district PCU supervisor:

‘Sometimes there’s overlap between roles, and families become confused who to call for which problems. Our caregivers have more training and work full-time, making them more reliable and knowledgeable, but this creates expectations that may exceed their actual competency.’

## Discussion

### Innovation as task integration, not task shifting

Full-time community caregivers at Ubolratana receive on average 9000 baht per month (US$1 ≈p33 THB), markedly higher than the 1000–2000 baht typically paid to VHVs for voluntary part-time work. The redefinition of community caregivers from part-time assistants and parallel CHWs into 24-hr members of a hospital-led local healthcare team represents a significant paradigm shift that addresses workforce shortages while maintaining cultural appropriateness (Perry et al. 2014). To mitigate burnout, the model emphasises caregiver pride, competency-based training, supervision, and embeddedness within home communities (Joshi et al. 2014). Critically, the Ubolratana caregivers operate as embedded members of the local team rather than as an independent CHW cadre, which preserves COC rather than adding a parallel service layer, a distinction we return to below in discussing the 2026 general-election campaign proposed ‘15000-baht community-nurse policy’. Outstanding service outcomes over 6 years and high home-based end-of-life-care uptake with high good death (FAMCARE) scores (Ooraikul et al. 2020) are best read as consequences of this integrated implementation.

### Outer-setting determinants: adaptive innovative financing, public trust, and the 15000-baht policy

The multi-source funding model is, on the one hand, a CFIR exemplar of innovation adaptability to a fragmented outer setting (Hanson et al. 2022). On the other hand, it should be read as an enabling and adaptive implementation strategy rather than as the entire innovation. The reliance on diverse sources (NHSO-managed UHC budgets, local government allocations, community-based donations, social-enterprise revenue, and private donations) underscores systemic inefficiencies in Thailand’s public health financing (Watabe et al. 2017, Patcharanarumol et al. 2018). Interviews also suggest that Ubolratana donation financing is built on public trust in the community hospital, not reliance only on a few private philanthropists. For example, Ubolratana’s inpatient private wing was built from community-wide donations of the district; residents saw it as an affordable investment if they need to be treated in the private ward that they donated. This history illustrates how the hospital director’s ‘social engineer’ role converted trust between the hospital and local residents into a community-financing resource. The dependence on such diverse and trust-mediated sources underscores the need for a more integrated national health financing framework to support community-based care models effectively.

Policy contents in the 2026 general-election campaigns echo this study’s findings: at least two major parties propose 15000-baht salaries for community nursing volunteers in all villages, and several parties propose upgrades to sustainable Thailand UHC (Policywatch 2026). A critical caveat from this study concerns implementation framing: if the 15000-baht policy creates a parallel cadre of independent community nurses rather than embedding them within hospital-led local teams as the Ubolratana model does, it risks deepening (not reducing), the very fragmentation that this model has overcome.

### Inner-setting determinants: local leadership as buildable capacity

Across our interviews, the single most consistently cited facilitator of both adoption and sustainment was sustained inner setting leadership: the long tenure of Apisit Thamrongwarangkun, the hospital culture that frames clinical staff as ‘social engineers’ for the district health system, and the continuity of this orientation under the next leadership generation. In CFIR terms, this fuses the high-level leaders construct (authority to allocate resources and shield innovation from short-term pressure) with the implementation lead/champion construct (day-to-day stewardship). The Ubolratana case therefore suggests that leadership is not transferred simply by idol copying, but can be built through mentorship, succession, institutional routines, and repeated community-facing practice. A PCU director articulated the implication for diffusion:

‘When other hospitals visit us and learn about our model, they often focus on the technical aspects, training curricula, funding mechanisms, and coordination systems. But they miss the cultural factors and community relationships that took years to develop. These can’t be easily transferred or replicated quickly.’

Districts seeking to adopt the model therefore need to audit not only its visible components but also the presence of these enabling inner setting and individual’s determinants, and they need deliberate leadership-development and succession mechanisms rather than assuming that technical components alone will diffuse.

### International comparisons through a CFIR lens

Three established community-based primary care programmes offer instructive comparators when read through CFIR. Brazil’s Family Health Strategy (Estratégia Saúde da Família, ESF) embeds Agentes Comunitários de Saúde (ACS) within multidisciplinary family health teams attached to a basic health unit (Macinko and Harris 2015). Like the Ubolratana model, the ACS are formal members of a hospital/clinic-led team rather than a parallel cadre, and Brazil’s experience suggests this integration is precisely what has allowed ESF to be sustained over three decades. The ACS sit primarily in the innovation and implementation process intersection, with strong outer-setting support (constitutional UHC and a federal funding line), highlighting that Ubolratana’s outer setting fragility is the dimension most in need of policy attention.

Ethiopia’s Health Extension Programme deploys salaried female health extension workers recruited from the communities they serve, with 1 year of pre-service training following completion of grade 10; the programme has scaled nationwide since its 2003 launch (Assefa et al. 2019). The health extension workers model is strong on outer setting (a coherent national policy and salary line) and on process (standardized selection and training), but weaker on inner-setting integration with referral hospitals than the Ubolratana model. In CFIR terms, Ubolratana trades national reach for stronger local integration; the policy question for Thailand is whether the 15000-baht proposal can deliver both.

Iran’s Behvarz programme, operated through village health houses linked to rural health centres for more than four decades, is closest to Ubolratana in its primary-care-anchored implementation process: Behvarz workers are recruited from their own rural communities (with priority to local candidates), undergo 2 years of residential pre-service training at District Behvarz training centres combining theoretical instruction with clinical placements in health houses and rural health centres, and receive ongoing in-service training from GPs at rural health centres that each oversee 5–6 village health houses (Javanparast et al. 2012). Iran’s experience confirms the Ubolratana finding that local recruitment together with clinic- and hospital-anchored training generate the trust and clinical competence required for sustainment. Compared with these three international programmes, Ubolratana’s distinctive vulnerability is not only financing but also the need to cultivate local hospital leadership and community trust in each adopting district; its recent leadership succession suggests that these capacities are buildable, but only through structured investment.

### CFIR-derived levers for change

Reading across the five CFIR domains, four cross-cutting determinants distinguish the Ubolratana experience and surface as practical ‘levers’ for districts seeking to learn from it: (i) sustained inner-setting leadership and a hospital culture that frames clinical staff as ‘social engineers’ (inner setting and individuals); (ii) deliberate adaptation of the innovation to local conditions (community-led caregiver selection, Buddhist-integrated spiritual care, and agricultural community banking), rather than off-the-shelf transfer (innovation and outer setting); (iii) a coordinated multi-level care process that engineers continuity through embedded roles rather than referral hand-offs (implementation process); and (iv) reflective evaluation through the COC centre that converts routine service data into second-loop learning and ongoing programme adjustment (implementation process: reflecting and evaluating). Districts seeking to adopt this model should therefore audit not only its visible components but the presence of these enabling determinants.

### What transfers and what must be cultivated: a transferability analysis

The question of whether the Ubolratana model is ‘replicable’ is, in our reading, best answered not as a binary but as a tiered analysis of its components. Drawing on the CFIR lens, we propose three tiers of transferability (Table 2). Tier 1 comprises directly transferable design elements, the 6-month competency-based curriculum and its delivery structure, the COC centre, the coordinated multi-level care process, the tax-deductibility donation channel, the iterative HbA1c quality-improvement loop, route-mapped daily caseloads, and standardized documentation for NHSO claims as an implementation-process tool. The three-stage community-led selection process straddles innovation and implementation process domains because it both defines the caregiver role and operationalizes engagement, context assessment, and tailoring. These structures, procedures, and instruments have been adopted with minimal modification in the 22-district scale-up under Ramathibodi Faculty of Medicine sponsorship, and we observed no in-principle barrier to their further diffusion.

**Table 2:** Transferability analysis of Ubolratana model components: what can be transferred directly, what must be locally adapted, and what requires deliberate cultivation.

Component	CFIR domain(s)	Implication for diffusion
Tier 1. Directly transferable design elements. These are standardized structures, curricula, and procedures that have been adopted with minimal modification in the 22-district scale-up under Ramathibodi Faculty of Medicine sponsorship^a^.		
720-hr caregiver training curriculum (6-month structure: weekday online theory plus Saturday supervised field practice; 300-baht daily stipend)	Innovation and implementation process	Standardized through partnership with a partner academic institution; the same curriculum has been deployed across the 22 scale-up districts.
Three-stage community-led caregiver selection (farmer test, public village assembly, multi-stakeholder endorsement)	Implementation process	Procedural, manualized and replicated in pilot and scale-up districts.
'Three doctors’ coordinated-care model	Implementation process	Independently tested in four provinces (Noree et al. 2022); transferable as a structural design.
COC centre structure	Inner setting	Adopted as a structural element in scale-up sites.
Tax-deductibility donation channel through hospital operating funds	Outer setting	A national policy mechanism approved during a former Minister of Public Health’s tenure; available to any public hospital.
Iterative quality-improvement loop targeting HbA1c 8%–9% cohort (‘most-movable-first’ triage)	Implementation process: reflecting and evaluating	Methodological, applicable to any community-based chronic-disease programme.
Route-mapped daily caseload (10–15 patients/day, clustered geographically)	Implementation process	Operational design, directly replicable.
Documentation practice enabling NHSO primary-care claims	Outer setting	Standard NHSO mechanism; transferable to any contracted facility.
Tier 2. Adaptable patterns requiring local instantiation. These are conceptual patterns whose specific instantiation must be found locally; the model offers a template, not a turnkey package.		
Academic-institution partnership for theoretical training	Outer setting and innovation	Other academic institutions with Ministry of Education-recognised nurse-aide curricula exist nationally; districts must identify and engage an analogous partner.
Section 35 corporate employment of persons with disabilities	Outer setting	The law applies to all public hospitals; each district must develop its own corporate partner relationships.
Private donor partnerships supporting caregiver salaries	Outer setting: partnerships and connections	Concept transferable; specific donors are not. Diffusion depends on each district finding its own philanthropic partners.
Buddhist temple-based hospice integration	Inner setting	Adaptable to districts with strong local religious or cultural institutions (Buddhist or analogous); local relationship-building required.
Agricultural community-banking mechanism (e.g. bamboo, bananas, organic produce)	Innovation and outer setting	Adaptable to whatever local crops or non-cash community assets are available.
Five-stream funding diversification approach	Innovation and outer setting	The diversification principle is transferable; the specific mix of streams must be locally constructed.
Tier 3. Capacities that require deliberate cultivation. These are time- and relationship-dependent capacities. They are not impossible to develop elsewhere, but they cannot be transferred as discrete components; they require sustained, structured investment, of the kind already embedded in the Ramathibodi 5-pillar programme (which explicitly includes workforce and education pillars). Successful diffusion in scale-up sites depends as much on whether these capacities are deliberately built as on whether Tier 1 components are adopted.		
Sustained, long-tenured ‘social engineer’ clinical leadership at the community-hospital level	Individuals: high-level leaders and implementation lead/champion	At Ubolratana district, this capacity was cultivated over more than a decade. For diffusion, the Ramathibodi 5-pillar programme’s workforce and education pillars are designed to develop equivalent leadership in scale-up sites; this is a multi-year deliberate investment rather than a one-off transfer.
Hospital organizational culture framing clinical staff as ‘social engineers’ for the district	Inner setting: culture and mission alignment	Builds top-down from clinical leadership; requires aligned recruitment, training, and continuous staff development. Diffusion requires explicit cultural-development workstreams in scale-up sites.
Multi-generational community trust and the social legitimacy of the caregiver role	Inner setting and individuals: innovation recipients	Accumulated incrementally through years of sustained engagement; cannot be shortcut. The three-stage selection process is a structural enabler of trust-building but does not by itself create trust.
Inter-hospital co-creator networks (peer directors across districts)	Outer setting: partnerships and connections	Built on personal relationships among community-hospital leaders. The Ramathibodi sponsorship now provides an institutional scaffold for such networks, but they remain person-to-person at the operational level.

^a^The 22-district scale-up refers to the Ramathibodi Faculty of Medicine sponsorship (2020–2024+) covering 22 districts in 15 provinces (53 villages). Implementation has been documented qualitatively; quantitative outcome evaluation across scale-up sites is ongoing.

Tier 2 comprises adaptable patterns whose specific instantiation must be found locally: the partnership with a private college offering a Ministry of Education-recognised nurse-aide curriculum, Section 35 corporate employment of persons with disabilities, donor partnerships supporting caregiver salaries, Buddhist temple-based hospice integration, the agricultural community-banking mechanism, and the broader five-stream funding diversification approach. These provide a template, not a turnkey package; each adopting district must construct its own equivalent partnerships and assemble its own funding mix from locally available streams.

Tier 3 comprises capacities that require deliberate cultivation rather than transfer: sustained, long-tenured ‘social engineer’ clinical leadership at the community-hospital level; leadership succession and mentorship; the corresponding hospital culture; multi-generational community trust in the caregiver role and in hospital-managed community financing; and inter-hospital co-creator networks. These are not impossible to develop elsewhere, but they cannot be deployed as discrete components; they require sustained, structured investment of the kind already embedded in the Ramathibodi 5-pillar programme’s workforce and education pillars. Successful diffusion in scale-up sites depends as much on whether these Tier 3 capacities are deliberately built as on whether Tier 1 components are adopted. The Ministry of Public Health, NHSO, and academic sponsors should therefore treat leadership-development pipelines, succession planning, and community-trust-building processes as first-class budget lines in any future scale-up programme, on a par with curriculum development and equipment procurement. We note that quantitative outcome evaluation across the 22 scale-up districts is ongoing; the tiered analysis above is consequently a hypothesis-generating framework that future longitudinal work should test.

### Limitations

Study limitations include: the qualitative design, limiting generalizability without adaptation; focus on one district that may not apply to different demographic, economic, or cultural settings; and the need for longitudinal studies to assess long-term sustainability and outcomes. The retrospective application of CFIR is additionally sensitive to recall and self-presentation bias, which we mitigated through multi-stakeholder triangulation across hospital administrators, caregivers, PCU personnel, local government, and community members. Future research should examine cost-effectiveness compared to traditional healthcare delivery, patient health outcomes and quality of life measures, scalability factors for diverse settings, and long-term financial sustainability assessment, including detailed cost analyses of benefit packages.

## Conclusions

The Ubolratana model presents an innovative and practical framework for 24-hr hospital-integrated community caregiving, supported by adaptive financing mechanisms. Read through a CFIR lens, its sustainment over 6 years rests on a tight coupling of inner-setting leadership, community-led implementation process design, enhanced caregiver competencies, and adaptive innovative financing, set against a fragmented outer setting that the model has (so far) successfully navigated rather than transformed. The NHSO should address these systemic constraints by developing integrated financing frameworks, clarifying oversight mechanisms post-decentralization, and implementing robust monitoring and evaluation systems for community-based care.

Above all, sustained local hospital leadership emerged as a critical CFIR-defined determinant of both adoption and sustainment, but the Ubolratana succession experience suggests that such leadership can be cultivated rather than treated as purely non-transferable. The model has been progressively scaled under Ramathibodi Faculty of Medicine sponsorship to 22 districts across 15 provinces (53 villages) over 2020–2024, indicating that diffusion is feasible; this diffusion, however, depends as much on building equivalent local champions, community trust, and hospital-led team integration as on transferring the technical components of the model. For policymakers and administrators, the Ubolratana experience offers practical strategies, and a structured way to read them through CFIR, for enhancing community-based care through local engagement and resource diversification.

## Data Availability

The data underlying this article cannot be shared publicly because they contain information that could compromise the privacy of research participants. De-identified data may be made available from the corresponding author upon reasonable request and subject to approval by the relevant ethics committee.

## Reflexivity statement

This qualitative study was conducted by a multidisciplinary research team with expertise in family medicine, palliative care, and community health and health systems research. Several members of the research team have professional experience within Thailand’s primary healthcare system, which provided contextual understanding of community-based care and health system governance. However, the researchers were not directly involved in the design, management, or implementation of the Ubolratana community caregiver programme. The research team remained attentive to potential positionality and interpretive bias throughout the research process. Semi-structured interview guides were collaboratively developed to ensure balanced exploration of both strengths and challenges of the Ubolratana model. During data collection, researchers encouraged participants to share diverse perspectives and experiences, including both positive outcomes and perceived limitations of the programme. Data analysis followed a reflexive thematic analysis approach. At least two researchers independently coded the interview transcripts and discussed discrepancies until consensus was reached. Regular analytical discussions were conducted within the research team to reflect on emerging interpretations and ensure that findings remained grounded in participants’ accounts rather than researchers’ prior assumptions. The multidisciplinary composition of the research team supported analytical triangulation and strengthened the credibility of the study findings.
